# A valid and reliable nutrition knowledge questionnaire for track and field athletes

**DOI:** 10.1186/s40795-017-0156-0

**Published:** 2017-04-12

**Authors:** Matthew James Walter Furber, Justin Dene Roberts, Michael George Roberts

**Affiliations:** 1 Department of Sport, University of Hertfordshire, School of Life & Medical Sciences, Health & Exercise Science, Hatfield, Hertfordshire AL10 9AB UK; 2 Department of Life Sciences, Anglia Ruskin University, Faculty of Science and Technology, Cambridge, CB1 1PT UK

**Keywords:** Nutrition knowledge, Athlete, Questionnaire, Validation, Sport nutrition

## Abstract

**Background:**

Establishing an understanding of an athlete’s nutrition knowledge can inform the coach/practitioner and support the development of the athlete. Thus the purpose of the study was to develop a psychometrically valid and reliable tool to assess general and sport nutrition knowledge.

**Methods:**

An 85 question questionnaire was developed in consultation with a panel of experts. Ninety-eight participants from the UK completed the questionnaire, and again 3 weeks later. The participants were classified into two groups: those with nutrition (NUT, *n* = 53) training (sport nutritionists and dietitians who were either practicing or undertaking a postgraduate qualification in the field), and those without (NONUT, *n* = 48) training (professionals and postgraduate students with no exposure to any form of nutrition training). The questionnaire was then administered to a pilot cohort of UK based track and field athletes (*n* = 59) who were requested to time how long it took to complete the questionnaire.

**Results:**

Psychometric statistical analysis of the results was completed, resulting in the removal of 23 questions for a total of 62 questions in the final questionnaire. The validated questionnaire was then administered to 58 track and field athletes. Internal consistency was assessed using Chronbach’s alpha (α > 0.7), Pearson’s correlation (*p* < 0.05) was used to assess reliability. Construct validity was evaluated using a *t*-test (*p* < 0.05). A total test retest correlation of 0.95 was achieved (sub-section range: 0.87–0.97). Internal consistency was accepted in each sub-section (α = 0.78–0.92) and the nutrition-trained group scored significantly higher on the overall questionnaire (80.4 vs 49.6%). The overall score for the athletic group was 61.0%.

**Conclusion:**

The questionnaire satisfied all psychometric measures and provides a new valid and reliable tool to assess general and sport nutrition knowledge of track and field athlete.

**Electronic supplementary material:**

The online version of this article (doi:10.1186/s40795-017-0156-0) contains supplementary material, which is available to authorized users.

## Background

Nutrition plays an important role in human health, it is postulated that nutrition is the most controllable risk factor impacting long-term health and chronic disease [1] and can be easily manipulated to improve exercise performance [2]. Consequently, optimal health and sport nutrition strategies have been subject to comprehensive research [3]. However recommendations may be controversial and can be misinterpreted, as such the sport and fitness industries are saturated with varying opinion, articles and internet material which can provide unsubstantiated claims [4]. Furthermore athletes’ diets are commonly reported as being nutritionally inadequate [5, 6], often in a negative energy balance and subsequent micronutrient deficiency and/or poor macronutrient choices [7]. The underlying reasons for this are unclear, but may include: 1) the athlete knows what to consume but does not do so; 2) the education messages given to the athlete are inaccurate; 3) the athlete is not getting educated in nutrition; 4) the athlete does not think nutrition is an important aspect of performance; and 5) the athlete thinks their nutrition habits are adequate. Additionally a number of social-economic factors may influence food choice [8]. Consequently when working with an athlete identifying the cause of the poor nutrition choices could ultimately lead to enhanced nutrition intake. The development of a valid and reliable tool to assess nutrition knowledge with the potential of (a) ruling out a knowledge issue or (b) having grounds for an intervention to address inadequate knowledge, would prove valuable.

To develop a valid and reliable instrument to measure psychological attributes a defined set of criteria needs to be met [9]. A predefined structure should be followed and a series of measures must be performed [9] to ensure questionnaire validity and reliability. A number of nutrition knowledge questionnaires have previously been developed using a range validation methods, targeting specific populations; New Zealand rugby coaches [10], South African adolescents [11], elderly [12] and inpatients [13], however the validity of the instrument is reduced if used in different populations.

Designing a valid and reliable tool to assess general and sport nutrition knowledge in an athletic population may provide the accurate information needed to advise better dietary choices and improve dietary intake [14]. A recent systematic review [15] highlighted 38 studies which have used a nutrition knowledge questionnaire, only one [14] of which met the full validity and reliability criteria. Furthermore with regards to comprehensiveness rating [15] the four questionnaires which scored highest on validity and reliability [14, 16–18] scored between 36 and 55% on comprehensiveness rating. There is a clear need for a psychometrically validated nutrition knowledge measure that can investigate the participant’s general and sport nutrition knowledge and the aims of this research were to develop a valid and reliable general and sport nutrition knowledge questionnaire for athletes.

## Methods

### Study design

The project was approved by the University of Hertfordshire Life and Medical Sciences ethics committee and was designed in two stages: the first to develop a new tool for measuring nutrition knowledge in athletes, the second to pilot the questionnaire in a random group of athletes to further determine validity.

**Fig. 1:**
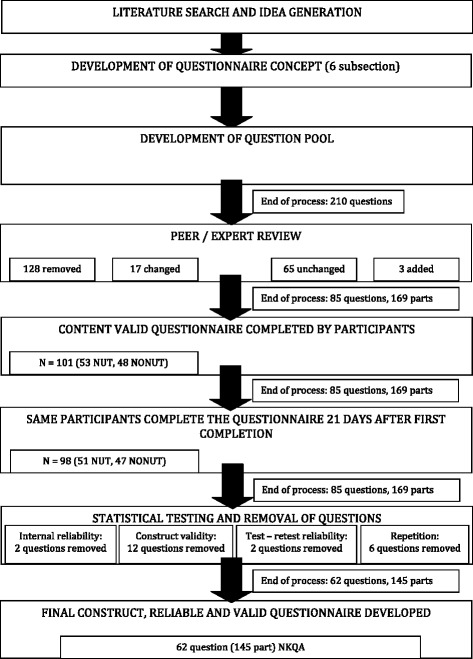
Schematic of processes completed to develop the NKQA

Eight separate processes were used to generate the final version of the Nutrition Knowledge Questionnaire for Athletes (NKQA) which is outlined in Fig. 1. The systematic process utalised in the development of the questionnaire measured for content validity, construct validity, test re-test reliability, internal consistency and test duration.

### Participants

#### Stage 1

Two population groups with differing exposure to nutritional training were selected to receive the questionnaire. The two groups were matched in education level, but with different professional expertise. The nutrition group (NUT) (*n* = 53) consisted of sport nutritionists and dietitians who were either practicing or undertaking a postgraduate qualification in the field. The non-nutrition group (NONUT) (*n* = 48) consisted of a range of professionals and postgraduate students with no exposure to any form of nutrition training (Table 1). Participants were recruited via the use of an email flyer and voluntarily contacted the research team to partake in the study.

**Table 1:** Participant characteristics

Characteristics	1st collection (*N* = 101)				2nd collection (*N* = 98)			
	NUT		NONUT		NUT		NONUT	
	n	%	n	%	n	%	n	%
Gender								
Male	31	64	26	54	30	59	26	55
Female	22	36	22	46	21	41	21	46

*NUT* Nutrition trained group, *NONUT* No nutrition training group

#### Stage 2

Following the development of the questionnaire it was administered to a cohort of UK track and field athletes (*n* = 59, Table 2). In reply to an advert/email athletes voluntarily contacted the research team to partake in the study.

**Table 2:** Athlete characteristics

	N	Age (years)	Weight (kg)	Height (cm)	National ranking
Athlete characteristics	59	24.8 ± 4.9	65.9 ± 9.8	173.5 ± 1.0	40 ± 29

*National ranking* Current national ranking in best event. (mean ± SD)

### Assessment of validity, reliability and statistical analysis

#### Content validity

The American College of Sports Medicine [19] position stand is an academic peer review publication capturing current literature and recommendations in the field of nutrition for athletes, thus it was adopted as a reference to construct the question answers from. Following a comprehensive literature search a pool of 210 questions were selected and the topics and subsections of the questionnaire were developed. The questions were either adapted from previous questionnaires or contrived from the use of literature searches and expert opinion. It was decided to have six definitive subsections within the questionnaire; carbohydrate, protein, fat, general nutrition, fluid and sports nutrition. These six areas were deemed important to assess as each impact either health, sporting performance or both. A meeting was held with 5 experts in the field (2 × graduate nutritionists - both with > 3 years experience working in elite sport, 2 × nutritionists - both Senior Lecturers, 1 × physiologist - Principal Lecturer) to discuss the merits of the questionnaire. Each question was read out loud by the lead researcher and was subsequently critiqued in a group discussion. Each question was reviewed for comprehension, relevance, accuracy, repetition and scientific support and was either ‘removed’, ‘changed’, or ‘left in’. *Construct Validity:* To demonstrate that the questionnaire differentiates nutrition knowledge, construct validity was assessed. The questionnaire was uploaded online and administered to the participants twice in a test retest manner, separated by 3 weeks. The period between questionnaire completion was considered long enough for the answers to be forgotten and short enough to minimise any real change in nutrition knowledge [9]. At the time of the initial administration the participants were not made aware of the second administration. The results from the first collection were used to assess internal consistency and construct validity; the data from both collection phases was used to assess reliability over time. A paired sample *t*-test was performed comparing the mean subsection and total scores achieved on the questionnaire from both the NUT and NONUT groups. A significance of *p* < 0.05 was selected. A Pearson’s Correlation was used to assess the correlation between the results from 98 participants who took the questionnaire at the two separate time points, a significance of *p* < 0.05 was selected. *Internal Consistency*: Each of the six subsections were assessed separately for internal consistency as each subsection was addressing a different area of knowledge. A Chronbach’s Alpha, with a minimum requirement of α > 0.7 was accepted to demonstrate sound internal consistency [9]. *Questionnaire Duration*: Following the statistical analysis of the questionnaire, the final copy was administered to a new cohort of participants. The participants completed the questionnaire and record how long it took to complete. Please see Additional file 1 document for the full version of the NQKA.

## Results

During the expert review 128 questions were removed, 17 had wording changes, 3 were added and 65 remained un-changed, resulting in an 85-question questionnaire. A number of questions included multiple parts, for example: Are the following foods high or low in carbohydrate: Beef, Lentils or Jelly babies? In total the 85 questions contained 145 parts. An example of a question which was removed in this process is: “To reduce your cholesterol do you think you should eat less: Cakes and biscuits, Skim milk, Ice Cream, the Fat on Meat, Sugar, Bread, Coconut, Avacodos.” This was removed on account of relevance, it was not deemed the question addressed the rational of the questionnaire.

The reliability and validity statistics for the final set of questions are displayed in Tables 3 and 4. Response for the retest phase of the study was high with, 51 of the original 53 (96%) participants from the NUT group, and 47 of the original 48 (98%) NONUT group completing the questionnaire for both test and retest phase. Three participants did not respond to the invitation to complete the questionnaire for a second time.

**Table 3:** The sub-section and total score achieved (mean, SD and percentage (%)) on the nutrition knowledge questionnaire for athlete by the NUT and NONUT groups

Nutrition knowledge sub-section (n)	NUT			NONUT		
	*Mean*	*s.d*	*Percentage (%)*	*Mean*	*s.d*	*Percentage (%)*
Carbohydrate (23)	19.1*	1.54	82.9	12.6	4.34	54.6
Protein (18)	13.9*	1.90	77.5	8.7	3.04	48.5
Fat (23)	19.5*	2.12	84.9	13.4	3.11	58.2
General nutrition (31)	25.7*	3.60	82.9	16.3	3.53	52.6
Fluid (15)	10.9*	2.28	72.8	6.0	2.81	40.0
Sports nutrition (35)	27.9*	5.67	78.5	14.9	4.46	42.2
Total (145)	116.7*	10.8	80.4	71.9	10.63	49.6

*NUT* Nutrition trained group, *NONUT* No nutrition training group

*Scored significantly higher within category than the NONUT (*p* < 0.001)

**Table 4:** Internal reliability of the nutrition knowledge questionnaire for athletes for the first data collection, also test retest reliability and identical response rate over two data collection periods separated by 3 weeks

Nutrition knowledge sub-section (n)	Internal reliability^a^	Test - retest correlation^b^	Identical responses from both tests (all) (%)	Identical responses from both tests (NUT) (%)	Identical responses from both tests NONUT) (%)
Carbohydrate (23)	.84	.95	91.0	94.5	87.5
Protein (18)	.81	.94	89.9	92.5	87.4
Fat (23)	.78	.97	93.5	93.4	93.6
General nutrition (31)	.86	.92	87.7	90.8	84.5
Fluid (15)	.82	.91	84.0	84.6	83.3
Sport nutrition (35)	.92	.87	83.4	84.5	82.4
Total (145)	N/A	.98	88.0	89.8	86.1

*NUT* Nutrition trained group, *NONUT* No nutrition training group

^a^Chronbach’s alpha

^b^Pearson’s correlation significant at < 0.05 (2-tailed)

The NUT group achieved a significantly (*p* < 0.05) higher score than the NONUT group in each of the subsections. In both groups the fat subsection was the highest scoring (84.9 and 58.2% in NUT and NONUT, respectively), whilst the lowest mean scores were observed in the fluid subsection (72.8 and 40.0% in NUT and NONUT, respectively). Overall the NUT group provided a correct answer for 80.4% of the questions, whereas the NONUT group answered less than half (49.6%) the questions correctly.

A strong test retest reliability (Table 4) was observed; the correlation for the total questionnaire was 0.98 (*p* < 0.05), all of the subsections produced a correlation ≥ 0.87 (*p <* 0.05). The internal reliability (Table 4) for each of the subsections achieves the psychometric requirements to determine reliability (Chronbach’s α > 0.7). The fat subsection produced the lowest alpha level at 0.78 and the sport nutrition subsection produced the highest alpha level at 0.92.

The test retest results for all participants produced an identical response to the same question 88.0% of the time, with the nutrition group exhibiting an identical response of 89.8% of the time and the NONUT group 86.1% of the time. The carbohydrate subsection produced the highest number of identical responses in the nutrition group with 94.5%, whereas the fat subsection produced the highest number of identical responses with 93.7% for the non-nutrition group. The sports nutrition subsection produced the lowest number of identical responses, with 84.5 and 82.4% for the nutrition and non-nutrition groups respectively.

The results of the NKQA completed by the athletes were consistent throughout the questionnaire. The athletes, on average, scored higher than NONUT group and lower than the NUT group in all subsections, with the percentage of total correct responses 61, 49.6 and 81.4% respectively (Table 5). On average the athletes scored lower than the total mean questionnaire score (61%) on both the fluid (53.8%) and sport nutrition (55.2%) subsections. The average completion time of the final questionnaire for the athletes was 15:20 ± 2:45 min.

**Table 5:** Sub-section and total score achieved (mean, SD and percentage (%)) on the nutrition knowledge questionnaire for athletes by the athletic population

Nutrition knowledge sub-section (n)	Athletes		
	Mean	*s.d*	*Percentage (%)*
Carbohydrate (23)	13.6	2.30	59.2
Protein (18)	11.9	2.70	66.0
Fat (23)	15.3	3.93	66.6
General nutrition (31)	19.3	3.68	62.2
Fluid (15)	8.1	2.81	53.8
Sports nutrition (35)	19.3	4.22	55.2
Total (145)	88.5	15.97	61.0

## Discussion

The aim of the present study was to develop a valid and reliable general and sport nutrition knowledge questionnaire which can be used as a practical tool to assess nutrition knowledge in track and field athletes.

The comprehensive and structured psychometric evaluation of the current questionnaire demonstrates strong reliability and validity. The NUT group (who had considerable training in the field of nutrition) produced > 30% (*p <* 0.05) more correct responses than the NONUT group (who had no prior exposure to nutrition training) throughout the questionnaire, such significant differences in the scores of the two groups is sufficient to assume construct validity [20]. Equally, the test retest correlation (0.98, *p* < 0.05) demonstrates satisfactory internal reliability [9] and internal consistency can also be assumed as each subsection produced a Chronbach’s alpha value > 0.7 [9], as such establishing a new tool for the assessment of general and sport nutrition knowledge in track and field athletes.

Furthermore the distribution of the questionnaire to an athletic population demonstrated that the athletes had greater nutrition knowledge (61.0%) than the NONUT cohort (49.6%), but less than NUT group (80.4%). It has previously been demonstrated that athletes’ nutrition knowledge is lower than that of a nutritionally educated population. Frederick and Hawkins [16] modified an existing 20-question nutrition knowledge questionnaire, 27 track athletes and 14 college students (nutrition major) completed the instrument scoring 77.9 and 87.3% respectively (*p* < 0.05). More recently Spendlove et al. [14] use a questionnaire developed by Parmenter et al., [21] to differentiate nutrition knowledge in elite Australian athletes (*n* 175) and a dietetic trained (*n* 53) cohort, scoring 57.6 and 86.2% respectively. Interestingly the results from the current study demonstrate that the lowest scoring subsection in the questionnaire for the athletes were fluid (53.8%) and sport nutrition (55.2%), which may be deemed as most specific to this population. However, relative to the results from the NONUT group in the athletes scored 13.8 and 13% higher in these sections, both greater than the mean score difference of 11.4% respectively. Similar trends in nutrition knowledge disparity between athletes and non-athletes have been observed [22, 23]. Furthermore, the difference in nutrition knowledge between the athletic population and nutrition trained group in this study are similar the previous research [14], providing further corroborating of the validity of the NKQA as a tool measure nutrition knowledge.

The length of the questionnaire is adequate to attain the relevant information needed to draw conclusions about the responders nutrition knowledge, but not too long to reduce compliance. It has been suggested that questionnaire length may impact participant response with longer questionnaire reducing compliance [24], however in a recent meta-analysis [25] only three of the 25 studies investigating questionnaire length demonstrated a weak correlation between questionnaire length and participant response. With 14 pages of questions and an average completion time of 15 min 20 s the questionnaire is of a moderate duration, however the test re-test completion rate of the questionnaire was high, with 98 of the original 101 participants completing the questionnaire on both occasions. Despite the questionnaire being longer than other nutrition knowledge questionnaires it is reasonable to suggest that the length will not impact completion and accuracy.

The test - retest correlation was high and consistent across all the subsections (r = 0.93 ± 0.04, range 0.87 – 0.97), and the total test - retest correlation for all 145 questions was 0.98. With such a strong test - retest reliability the questionnaire provides a tool which if administered over time (>3 weeks between administrations) can be used to assess the effectiveness of an intervention or nutrition education programme.

An increased inability to answer the questions correctly can increase the respondent bias [26], reducing the accuracy of the questionnaire. To control for this the questionnaire included a briefing paragraph detailing the importance of not guessing, also the presence of an ‘unsure’ answer choice provided the responder with an answer option. The results from both groups within this study produced a higher number of identical responses across the test - retest period (89.8 vs 86.1%; NUT vs. NONUT) thus it is fair to assume that the respondent bias of the questionnaire was low and indicates a low percentage of questions with a guessed answer. A slightly lower test re-test identical question response was observed in the NONUT group relative to the NUT group, which could be attributed to a lower knowledge, less confident and more indecisiveness in the answers chosen [26]. However the NONUT group still produced a very high identical response rate of 86%, as such this is of little concern to the validity and reliability of the questionnaire.

Previously the most comprehensive psychometrically validated sport nutrition knowledge questionnaire developed [10] lacked a broad general nutrition subsection, raising concerns over conclusions drawn about the participants’ general nutrition knowledge. The current questionnaire includes 32 questions (95 parts) addressing macronutrients and general nutrition, as such the range and quantity of these questions are similar to that of a previously validated general nutrition knowledge questionnaire for adults [21], additionally a further 33 questions (50 parts) address hydration and sport specific nutrition, thus provide sufficient information for conclusions to be drawn about the specific areas of the individuals general and or sport nutrition knowledge. Furthermore using the rating scores outlined in a recent systematic review [15] the current study meets all the criteria required to confirm validity and reliability, and is only the second questionnaire to do so [14]. Also the current questionnaire would score around 9 – 10 out 11 (81 – 91%) on the comprehensiveness rating, outscoring the other valid and reliable questionnaire [14] by ~5 points (~45%). Therefore it is fair to assume the range of subsections represents a greater depth of assessment than any current available valid and reliable tool.

A systematic review from Heaney et al. [5] found a weak positive association (*r* < 0.44) between nutrition knowledge and improved dietary intake in athletic populations, and the evidence supporting was inconclusive, as such the relationship between enhanced nutrition knowledge and improved dietary choices is unclear. Furthermore, the validity of the sport specific nutrition knowledge assessment was inconsistent within these studies. Importantly, nutrition opinion can be varied and it is common for practitioners to have differing thoughts on what optimum nutrition is and how this can be achieved. The questions and answers within this questionnaire were developed and calibrated against peer reviewed position statements [19] however must be interpreted but the practitioner. The questionnaire should be used as a tool to investigate the knowledge of the athlete, not as a complete solution to comprehend nutrition knowledge and dietary intake of the responder.

It is well established that socio-economic class can play an important role in relation to nutrition knowledge and dietary practice/supplement usage [8]. A limitation to this questionnaire was that socio-economic class was not captured, if the questionnaire is to be used to compare knowledge scores of different athletes, consideration should be paid to the socio-economic difference of the individuals. Furthermore the author’s recgonise that some areas of nutrition knowledge are not addressed within the questionnaire therefore conclusions can only be drawn from the questions selected. In addition to this, some of the questions (event specific nutrition), may not be relevant to all athletes and consideration should be made with the interpretation of these questions (e.g., question 50 and 61).

To date this questionnaire is the most robust measure of nutrition knowledge in track and field athletes; however it is important to note that this questionnaire was developed to assess the knowledge of UK Track and Field athletes. If used for a different athletic population the questionnaire is reduced in validity, as such if this NQKA is selected to be use in a different athletic population it would be recommended to make subtle, relevant changes to the questions and test the validity prior to use.

## Conclusion

The NKQA provides a psychometrically validated and reliable tool in the assessment of general and sport nutrition knowledge in track and field athletes. The included questions cover a broad range of general and sport nutrition topics consequently the differentiation in nutrition knowledge subsections are distinguishable from the results. As such the NKQA and should provide a quick, valid and reliable tool to assess an nutrition knowledge.

## Additional file

Additional file 1: General and Sport Nutrition Knowledge Questionnaire. (DOCX 377 kb)

## Abbreviations

NKQA: Nutrition Knowledge Questionnaire for Athletes
NONUT: Group with no nutrition training
NUT: Group with nutrition training
