# Supplementary material for: A valid and reliable nutrition knowledge questionnaire for track and field athletes
Source: BMC Nutr. 2017 Apr 12;3:36. doi: 10.1186/s40795-017-0156-0 (PMC7050860; doi:10.1186/s40795-017-0156-0)
Supplement: Supplementary file 1 — General and Sport Nutrition Knowledge Questionnaire. (DOCX 377 kb) [file 40795_2017_156_MOESM1_ESM.docx]

General and Sport Nutrition Knowledge Questionnaire

Fill out the questions below to the best of your knowledge. Please do not guess, if you are unsure of an answer please tick the ‘UNSURE’ box.

CARBOHYDRATE

1. In general are these foods High or Low in carbohydrate? *(Please tick one box per food).*

High Low Unsure

Beef

Pasta

Cabbage

Weetabix

Chocolate spread

Lentils

Wholemeal bread

Chicken

Jelly babies

1. In general as an athlete, what percentage of your diet should be made up from carbohydrate? *(Please tick one box)*

10 - 25 %

25 - 40 %

50 - 65 %

65 - 80 %

Unsure

1. Do you know what the glycemic index is?

Yes No

*If you answer no, please go to question 7.*

1. Which of the following phrases best describes the glycemic index? *(Please tick one box).*

The amount of carbohydrate a food contains

The extent to which carbohydrate food raises blood sugar levels

The extent to which protein food raises blood sugar levels

The extent to which carbohydrate food raises blood pressure

Unsure

1. In general is it best to eat a diet rich in High or Low glycemic index carbohydrates *(Please tick one box).*

High Low Unsure

1. Which of these foods are classified as High or Low in the glycemic index? *(Please tick one box per food).*

High Low Unsure

Porridge oats

Chick Peas

Sweets (e.g. Gummi Bears)

Dark chocolate (>70%coca)

Honey

Peanut butter

Basmati rice

Jacket potato

1. Brown sugar is a healthier alternative the white sugar *(Please tick one box)*

True False Unsure

1. A high carbohydrate diet helps to reduce protein breakdown in the body.

Agree Disagree Unsure

PROTEIN

1. Would you agree or disagree with the following statements? (*Tick one box per question)*.
   1. When lifting heavy weights the body uses protein as its main energy source.

Agree Disagree Unsure

- 1. Chicken is a very good source of energy to help fuel high intensity exercise.

Agree Disagree Unsure

1. Are the following foods High or Low in protein? *(Tick one box per food)*

High Low Unsure

Chicken

Kidney beans

Potato

Spaghetti

Tuna

Egg

Peanut butter

Spinach

Cornflakes

Malt Loaf

1. What is the main use for protein in the body? (*Please tick one box)*.

Energy Source

Growth and repair

Improve hydration

All of the above

Unsure

1. How much protein is there in the following food items? *(please tick one box per question)*

1 pint of skimmed milk

0.6 grams 6 grams 12 grams 40 grams Unsure

250 g tin of tuna

10 grams 20 grams 35 grams 50 grams Unsure

1 slice of white bread

0 grams 2 grams 4 grams 8 grams Unsure

1. In general how much protein (grams) should an average 70 kg male athlete eat per day? *(Please tick one box)*

49 – 77 grams

105 – 119 grams

154 – 196 grams

217 – 266 grams

Unsure

1. There is more protein in a glass of whole milk compared to skimmed milk? (*True or false?).*

True False Unsure

FAT

1. For improvements in health, what type of fat do experts recommend should be reduced in the diet? *(Please tick one box*).

Monosaturated fat

Polyunsaturated fat

Saturated fat

Unsure

1. Are these foods high in saturated or poly-unsaturated fat *(Please tick one box per food)?*

Saturated Poly - unsaturated Unsure

Pumpkin Seeds

Pork Chop

Butter

Olive Oil

Mackerel

Packet of Crisps

1. Which of the following lipoproteins increase cholesterol? (HDL = high density lipoprotein) or (LDL = low density lipoprotein) (*Please tick one box)*

HDL’s

LDL’s

Unsure

1. The following foods are high in cholesterol. (*Please tick one box per item)*

True False Unsure

Coconut Oil

Red Meat

Eggs

Walnuts

Whole Milk

Sunflower Seeds

Sardines

1. Do you think these foods are High or Low in fat? *(Please tick one box per item).*

High Low Unsure

Orange

Avocado

Cottage cheese

White bread

Peanuts

Sunflower seeds

Jam

Banana

VITAMINS AND MINERALS

1. Do you agree or disagree with the following statements *(Please tick one box per question)*
   1. 500 ml of orange juice has the same number of calories as 500ml of orange squash.

Agree Disagree Unsure

- 1. There is more calcium in a glass of whole milk than skimmed milk.

True False Unsure

1. What is the role of antioxidants in the body? (*Please tick one box)*

Help with energy production

Help prevent against cell damage

Increase metabolic rate

Improve hydration status

Unsure

1. In General the following foods are naturally rich in antioxidant. *(True or false).*

True False Unsure

Red Meat

White Fish

Fruit

Vegetables

Dairy

Unsure

1. If you want to eat something that is rich in Vitamin C, which of the following foods would you eat? (*Please tick one box per question*)
   1. Oranges Beef Unsure
   2. Red pepper Spinach Unsure
   3. Whitebait Baked beans Unsure
2. If you want to eat something that is rich in iron, which of the following foods would you eat? (*Please tick one box per question*)
   1. Spinach Milk Unsure
   2. Chickpeas Banana Unsure
   3. All Bran Yoghurt Unsure
3. If you want to eat something that is rich in calcium, which of the following foods would you eat? (*Please tick one box per question*)
   1. Spinach Chicken Unsure
   2. Skimmed Milk Sunflower seeds Unsure
   3. Potato Cheddar cheese Unsure
4. Which cooking method is best to help maintain the vitamin and minerals content within vegetables. (*Please tick one box)*.

Steam

Boil

Fry

Grill

Unsure

1. B vitamins are important for exercise because they help with: (*Please tick one box)*.

Hydration status

Energy Production

Immunity

All of the above

Unsure

GENERAL NUTRITION

1. Cutting out 10 grams of carbohydrate from your diet will result in greater weight loss than cutting out 10 grams of fat. (*Please tick one box)*.

True False Unsure

1. What food category are vegetables classified in? (Such as cabbage, carrots, onions, peppers, courgettes etc) (*Please tick one box).*

Carbohydrate

Protein

Fat

Unsure

1. During resting conditions, which is the predominant energy source the body uses? *(Please tick one box)*.

Carbohydrate

Protein

Fat

Unsure

1. How many calories are there in 1 gram of each of the following macronutrients and Alcohol? (*Please tick one box)*

Carbohydrate Protein Fat Alcohol

2 kcal

4 kcal

7 kcal

9 kcal

13 kcal

Unsure

1. Does one glass of orange squash count towards the government recommended ‘5 a day’? (*Please tick one box)*

Yes

No

Unsure

1. If you are trying to lose weight and want to have a snack what would be best the best food to snack on? (*Please tick one box per question)*.
   1. Peanut butter on a bagel or Tuna sandwich
   2. 100 g chicken wrap or 100 g pork pie
   3. 150 g Tomato salad or 100 g rice pudding
   4. Cottage cheese and berries or Cheddar Cheese and crackers

(50 grams) (50 grams

Fluid

1. In general, how much fluid should you drink on average per day? (Please tick one box)

0 – 0.99 litres

1 – 1.99 litres

2 – 2.99 litres

3 – 3.99 litres

4 – 4.99 litres

Unsure

1. During exercise greater than one hour, what are the current guidelines for fluid consumption? *(Please tick one box).*

0 – 200 ml

200 – 400 ml

400 – 800 ml

800 – 1,200 ml

1,200 ml +

1. In general, at what percentage of body dehydration would you start to see a decrease in exercise performance? (*Please tick one box).*

0.5 %

2 %

4 %

6 %

8 %

Unsure

1. A small amount of sodium added to fluid will help increase water absorption and improve hydration status? (*Please tick one box).*

True False Unsure

1. In the first 2 hours after exercise how much fluid should you aim to drink?

10 % of sweat loss

50 % of sweat loss

75 % of sweat loss

100 % of sweat loss

150 % of sweat loss

1. What type of drink would be best to consume in the following situations? *(Please tick one box per situation).*

Hypotonic Isotonic Hypertonic Unsure

1. Exercise lasting 0 – 45 minutes
2. Exercise lasting 45 – 90 minutes
3. Post exercise
4. How much carbohydrate is there in an isotonic sports drink? *(Please tick one box).*

0 – 3 %

4 – 8 %

8 – 11 %

12 – 15 %

Unsure

1. The following drinks are isotonic. *(Please tick one box per question)*

True False Unsure

1. Lucozade Sport
2. Gatorade
3. Red Bull
4. Water
5. Forgoodness Shakes

SPORTING PERFORMANCE AND SUPPLEMENTATION

*Please note, if you have not heard of any of the supplements listed below or are unsure of how they work, please tick the UNSURE box.*

1. A high protein meal 1 hour before competing in a power event is recommended to enhance performance. *(Please tick one box)*

True False Unsure

1. A high carbohydrate meal 2 – 4 hours pre exercise can lead to improvements in endurance performance. *(True or false?)*

True False Unsure

1. Which of the following drinks contains the highest amount of carbohydrate? (P*lease Tick one box)*

500 ml Coke

500 ml Powerade

500 ml full fat milk

500 ml orange squash

Unsure

1. In general, which of the following meals would be recommended to eat 3 hours before training? (P*lease Tick one box per question).*
   1. Steak and chips or Cous cous and tuna
   2. Mars bar and crisps or Bread and jam sandwich
   3. Chicken sandwich or Cornish Pasty
   4. Pasta and pesto or Green leaf salad
2. In general, what would be the best item to snack on in the 30 minutes pre exercise? *(Please tick one box per question).*
   1. Jelly babies or Peanuts
   2. Chocolate or Banana
   3. Sunflower seeds or ½ a white bread jam sandwich
   4. Cereal Bar or 50g low fat crisps
3. For a power athlete trying to increase muscle mass how much protein should they be eating per day (g/kg BM = grams per kilogram of body mass). *(Please tick one box)*.

0.5 – 0.9 g.kg BM

1.0 – 1.4 g.kg BM

1.5 – 2.0 g.kg BM

2.1 – 2.5 g.kg BM

2.6 – 3.0 g.kg BM

Unsure

1. If using a whey protein supplement, how much protein (in grams) do guidelines state should be consumed in one serving? *(Please tick one box)*.

10 – 17 g

18 – 24 g

25 – 30 g

31 - 37 g

38 – 45g

Unsure

1. If competing twice in one day, morning and evening:
   1. When is the optimum time to eat after the first event? (*Please tick one box*).

0 – 45 minutes

45 – 90 minutes

90 – 125 minutes

Eat after the second race.

Unsure

- 1. Is it more important to replace carbohydrate, protein or fat after the first event? *(Please tick one box)*.

Carbohydrate

Protein

Fat

Unsure

1. When carbohydrate loading, what percentage of your diet should come from carbohydrate? *(Please tick one box)*.

45%

60%

75%

90%

Unsure

1. Ideally, what percentage body fat would a world-class athlete have in the following events? *(Please tick one box per discipline)*

Sprinter Long Jumper Marathon Runner

0 – 3 % 0 – 3 % 0 – 3 %

4 – 10 % 4 – 10 % 4 – 10 %

11 – 14 % 11 – 14 % 11 – 14 %

15 – 20 % 15 – 20 % 15 – 20 %

Unsure Unsure Unsure

1. Is it always beneficial to have a isotonic sports drink in the 60 minutes pre event? *(Please tick one box)*

Yes

No

Unsure

1. What food group is made up of amino acids? *(Please tick one box)*

Carbohydrate

Protein

Fat

Unsure

1. If an amino acid is termed ‘essential’ what does this mean? *(Please tick one box)*

You should consume this in your diet

It is produced in the body

Should eat it before you exercise

Unsure

1. Immediately post exercise is it best to consume high or low glycemic index carbohydrates to support muscle glycogen recovery? *(Please tick one box)*.

High GI

Low GI

Unsure

1. Salt tablets could be used as a preventative if you regularly suffer from cramp during exercise. *(Please tick one box)*

Agree Disagree Unsure

1. Supplementing with caffeine will result in a decrease in performance in the following events? (*Tick as many as relevant)*.

True False Unsure

Sprinting

Long Jump

800 m

Shot Putt

Marathon

All or the above

1. Supplementing creatine has the same effect as supplementing with whey protein (*Please tick one box)*.

True False Unsure

1. In general, which athletic group would benefit from supplementing creatine monohydrate? (*Tick as many as relevant)*.

Speed and power

Endurance

Unsure

1. If using creatine how much is recommended you should take per day? (*Please tick one box)*.

1 gram

3 grams

5 grams

7 grams

9 grams

Unsure

1. Which type of athlete, has research has shown, could benefit from taking sodium bicarbonate as a performance aid? *(Please tick one box)*

100 – 200 meters

400 – 1500 meters

5,000 – Marathon

Throwers

Unsure

1. What are the physiological benefits of taking sodium bicarbonate? *(Please tick one box)*

Increase energy production

Increase free radical removal

Improve hydration status

Maintain blood pH

Increase maximal voluntary contraction

Unsure
